# Prognostic Value of Prerevascularization Fractional Flow Reserve Mediated by the Postrevascularization Level

**DOI:** 10.1001/jamanetworkopen.2020.18162

**Published:** 2020-09-30

**Authors:** Rikuta Hamaya, Murray A. Mittleman, Masahiro Hoshino, Yoshihisa Kanaji, Tadashi Murai, Joo Myung Lee, Ki Hong Choi, Jun-Jie Zhang, Fei Ye, Xiaobo Li, Zhen Ge, Shao-Liang Chen, Tsunekazu Kakuta

**Affiliations:** 1 Division of Cardiovascular Medicine, Tsuchiura Kyodo General Hospital, Ibaraki, Japan; 2 Department of Epidemiology, Harvard T.H. Chan School of Public Health, Boston, Massachusetts; 3 Cardiovascular Epidemiology Research Unit, Beth Israel Deaconess Medical Center, Boston, Massachusetts; 4 Harvard Medical School, Boston, Massachusetts; 5 Division of Cardiology, Department of Internal Medicine, Heart Vascular Stroke Institute, Samsung Medical Center, Sungkyunkwan University School of Medicine, Seoul, Korea; 6 Division of Cardiology, Nanjing First Hospital, Nanjing Medical University, Nanjing, China

## Abstract

**Importance:**

The prognostic value of pre–percutaneous coronary intervention (PCI) fractional flow reserve (FFR) may be associated with the post-PCI FFR and their interaction. To correctly interpret the prognostic value of pre-PCI FFR, it is essential to understand to what extent the association of pre-PCI FFR with clinical outcomes is explained by post-PCI FFR.

**Objective:**

To investigate the extent to which post-PCI FFR mediates the association of pre-PCI FFR with vessel-related outcomes using an international, multicenter collaboration registry.

**Design, Setting, and Participants:**

This cohort study used pooled patient data from 4 international FFR registries. A total of 1488 patients with pre-PCI FFR of 0.80 or less who underwent elective PCI were included. Data collection was conducted from November 2011 to August 2019, and analysis was conducted from September 2019 to July 2020.

**Main Outcomes and Measures:**

The primary outcome was target vessel failure (TVF) during 2 years of follow-up. The extent to which post-PCI FFR of less than 0.90 mediated the association of pre-PCI FFR less than 0.75 (vs pre-PCI FFR of 0.75 or greater) with TVF was evaluated using a mediation analysis in a counterfactual framework.

**Results:**

Among 1488 patients, the mean (SD) age was 63.5 (9.9) years and 1161 patients (78.0%) were men. The median (interquartile range) pre-PCI and post-PCI FFR were 0.71 (0.62-0.76) and 0.88 (0.83-0.92), respectively. The direct association of low pre-PCI FFR (ie, <0.75) with TVF was significant (odds ratio, 1.81; 95% CI, 1.03-3.17; *P* = .04), while the mediation by post-PCI FFR level of less than 0.90 was not (indirect association: odds ratio, 1.03; 95% CI, 0.98-1.09; *P* = .24). In sensitivity analyses using several pre-PCI cutoffs, the mediations by post-PCI FFR were consistently weak.

**Conclusions and Relevance:**

In this study, the association of pre-PCI FFR with TVF was not significantly mediated by post-PCI FFR. Poor prognosis due to progressed atherosclerosis, represented as low FFR, may not be reversed by successful PCI that increases FFR. Therefore, the prognostic value of pre-PCI FFR may mainly reflect the global atherosclerotic burden, not the extent of the modifiable epicardial stenosis.

## Key Points

**Question:**

To what extent is the association of pre–percutaneous coronary intervention (PCI) fractional flow reserve (FFR) with clinical outcomes explained by the association of pre-PCI FFR with post-PCI FFR?

**Findings:**

In this cohort study of 1488 patients, low pre-PCI FFR was significantly associated with target vessel failure but not mediated by post-PCI FFR.

**Meaning:**

The results of this study suggest that the prognostic information of pre-PCI FFR may not be associated with the results of PCI, represented as post-PCI FFR, indicating that the prognostic information of pre-PCI FFR may mainly reflect the global atherosclerotic burden of the artery, not the extent of the modifiable epicardial stenosis.

## Teaser

This cohort study investigates the extent to which post–percutaneous coronary intervention (PCI) fractional flow reserve (FFR) mediates the association of pre-PCI FFR with vessel-related outcomes using an international, multicenter collaboration registry.

## Introduction

Fractional flow reserve (FFR) has become an established marker of epicardial stenosis severity and is often used for guiding coronary revascularization. Previous studies have consistently demonstrated the association between FFR and future adverse events, and this is another important property of FFR.^1,2,3^ When percutaneous coronary intervention (PCI) is indicated, FFR may be assessed on 2 occasions, pre-PCI and post-PCI, and in either timing, low FFR is associated with a higher incidence of future adverse events.^1,2,3^ Potential explanations for theses associations are that pre-PCI FFR might reflect not only the regional ischemia but also global atherosclerotic burden, and post-PCI FFR could implicate the residual risk of suboptimal stenting in addition to residual diffuse coronary artery disease after successful PCI.^2,3,4^

Given that post-PCI FFR, a major surrogate of future event risk,^1,2,5^ can be partially determined by the pre-PCI FFR, the prognostic value of pre-PCI FFR may depend on the FFR achieved by PCI. To correctly interpret the prognostic information of pre-PCI FFR, we need to better understand to what extent the association of pre-PCI FFR with clinical outcomes is explained by post-PCI FFR.^6^ However, to our knowledge, there has been no study that has directly addressed this question. Therefore, we investigated the extent to which post-PCI FFR mediates the association of pre-PCI FFR with vessel-related outcomes using an international, multicenter collaboration registry.

## Methods

### Patient Population

The study population was from the International Post PCI FFR registry, which included 4 different registries from Japan, Korea, and China. All patients in this registry underwent second-generation drug eluting stent implantation and had clinical, angiographic, and poststent FFR data available. The 3V-FFR-FRIENDS registry (NCT01621438) enrolled 1136 patients who underwent 3-vessel FFR measurements from 12 centers in Korea, Japan, and China, starting in November 2011 with 2 years of follow-up and analysis reported in August 2017.^7^ Of this study, 266 patients (with 337 vessels) with poststent FFR measurement were included in the present registry. The COE-PERSPECTIVE registry (NCT01873560) was designed to evaluate the clinical relevance of poststenting FFR and enrolled a total 835 patients from 9 hospitals in Korea and Japan who underwent poststenting FFR assessment after angiographically successful PCI. This study started in May 2013, had 2 years of follow-up, and was reported in August 2019.^8^ The institutional registry of Tsuchiura Kyodo General Hospital, Ibaraki, Japan included 347 patients (with 357 vessels) who underwent post-PCI FFR measurement. This study started in January 2011, with 2.4 years of follow-up, and was reported in January 2018.^9^ The DKCRUSH VII registry (ChiCTR-PRCH-12001976) enrolled 1476 patients from 9 hospitals to evaluate the prognostic value of poststenting FFR. This study started in May 2012, with 3 years of follow-up, and was reported in February 2017.^10^ The data of 780 patients from Nanjing First Hospital were included in this registry. The international Post PCI FFR registry included a total of 2228 patients with 2425 vessels. In the present study, we analyzed 1488 patients whose pre-PCI FFR values were available and less than or equal to 0.80, with no missing covariates. Each study’s protocol was approved by the institutional review board or ethics committee at each participating center, and all patients provided written informed consent. The study protocols were in accordance with the Declaration of Helsinki.^11^ The study protocol was registered in clinicaltrials.gov (NCT04012281) and approved by the Tsuchiura Kyodo General Hospital institutional review board. This report follows the Strengthening the Reporting of Observational Studies in Epidemiology (STROBE) reporting guideline.

### Physiological Studies

We obtained FFR using a single pressure-sensor tipped intracoronary wire (Volcano or St Jude Medical). After calibration and equalization to aortic pressure, it was placed at the distal segment of the target vessel. A continuous infusion of adenosine or adenosine triphosphate was used in most patients for inducing hyperemia. Intracoronary administration of papaverine, nicorandil, or adenosine was used in 182 of 2200 patients (8.3%) in this registry. FFR was defined as the ratio of distal to aortic pressure at hyperemia.

### Definition of Low FFR

We a priori defined 2 combinations of exposure (low pre-PCI FFR) and potential mediator (low post-PCI FFR) in line with previous studies. First, we compared low pre-PCI FFR (defined as FFR <0.75) with gray-zone FFR (FFR, 0.75-0.80)^12^; the corresponding post-PCI FFR was regarded as low if less than 0.90 according to a meta-analysis of 105 studies.^5^ Second, we set low pre-PCI FFR as less than 0.67 (as a proposed more strict cutoff for revascularizaion^2^) and the corresponding low post-PCI FFR as 0.80 or less, indicating an unsuccessful PCI. Given that there is no consensus regarding the combination of pre-PCI FFR and post-PCI FFR thresholds, sensitivity analyses were conducted as follows: (1) examining several cutoffs of pre-PCI FFR for defining the exposure and (2) using continuous post-PCI FFR as a mediator.

### Patient Care and Clinical Follow-up

The primary clinical outcome for our analysis was target vessel failure (TVF), defined as a composite of death from cardiovascular causes, target vessel–related myocardial infarction (MI), and clinically driven target vessel revascularization during 2-year follow-up. All deaths were considered cardiovascular unless an undisputed noncardiovascular cause was present. Clinically driven revascularization was defined as repeat revascularization in the presence of diameter stenosis percentage of 70 or greater or 50 or greater with at least 1 of the following: (1) recurrence of anginal symptoms; (2) positive noninvasive test; or (3) positive invasive physiologic test.

### Mediation Analysis

**Figure 1.**
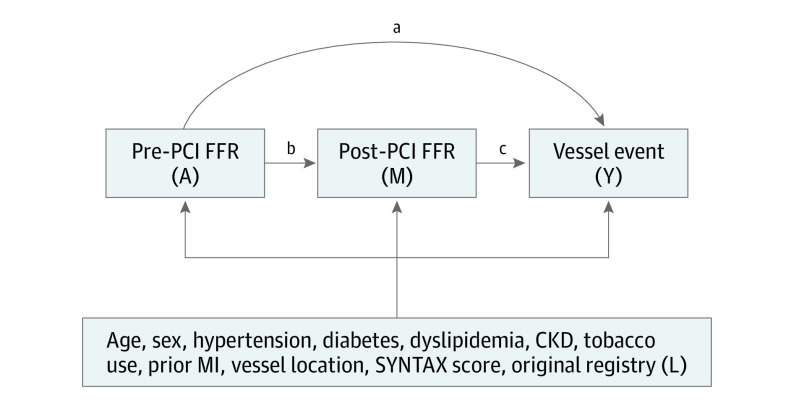
Proposed Mediational Pathway of This Study Arrows indicate the flow of association. *A* (exposure) is pre-percutaneous coronary intervention (PCI) fractional flow reserve (FFR), the origin of the association in which we are interested in. *M* (mediator), post-PCI FFR, is a variable that may modify the exposure-outcome association. *Y* (outcome) is target vessel failure (TVF), a primary end point of this study. *L* represents a set of confounders. Pathways *a* (effect of low pre-PCI FFR on TVF if effect of pre-PCI FFR on post-PCI FFR were blocked) and *bc* (low pre-PCI FFR on TVF when controlling pre-PCI FFR and changing post-PCI FFR status from the level it would have been at low pre-PCI FFR) indicate the direct and indirect pathway from the exposure to the outcome, respectively. CKD indicates chronic kidney disease; and MI, myocardial infarction.

Our conceptual model is summarized in Figure 1. We set the exposure, *A*, as pre-PCI FFR; the outcome, *Y*, as 2-year TVF; the mediator, *M* as post-PCI FFR; and the confounders, *L*, as age (years, continuous), sex (male or female), hypertension (yes or no), diabetes (yes or no), dyslipidemia (yes or no), chronic kidney disease (yes or no), tobacco use (ever or never), prior MI (yes or no), vessel location (left anterior descending coronary artery, left circumflex artery, or right coronary artery), pre-PCI SYNTAX score (quartile), and the original registry (4 categories). Univariable and multivariable logistic regression models were used to evaluate the association between the following: (1) exposure and outcome, (2) mediator and outcome, and (3) exposure and mediator. We tested the mediatory association of post-PCI FFR on the association between pre-PCI FFR and TVF by using a mediation analysis based on a counterfactual framework using methods developed by VanderWeele et al.^6^ This mediation analysis framework has 4 key assumptions, as follows: (1) no unmeasured exposure-outcome confounding after conditioning on the measured confounders; (2) no unmeasured mediator-outcome confounding after conditioning on exposure and the measured confounders; (3) no unmeasured exposure-mediator confounding after conditioning on the measured confounders; and (4) no mediator-outcome confounding affected by the exposure. Under these assumptions, the natural direct effect (NDE, represented by pathway *a* in Figure 1) represents the average effect of low pre-PCI FFR on TVF if the effect of pre-PCI FFR on post-PCI FFR had been blocked such that post-PCI FFR would remain if pre-PCI FFR had not been low. The natural indirect effect (NIE, represented by pathways *b* and *c* in Figure 1) represents the estimated effect of low pre-PCI FFR on TVF when controlling for pre-PCI FFR while changing post-PCI FFR status from the level it would have been at low pre-PCI FFR. Total effect (TE) indicates the overall effect of exposure on outcome. We used robust variance estimation to obtain 95% CIs and *P* values. The proportion of the association between pre-PCI FFR and TVF that is mediated through post-PCI FFR was estimated by dividing the logistic regression coefficient for the NIE by the coefficient for the TE.^6^

### Statistical Analysis

Data were analyzed on a per-patient basis for clinical characteristics and on a per-vessel basis for comparison of lesion characteristics, including physiological studies and vessel-related clinical outcomes. Categorical data, expressed as frequencies and percentages, were compared using the χ^2^ test or Fisher exact test, as appropriate. Continuous biochemical or physiological data are shown as mean (SD) or median (interquartile range [IQR]) and were analyzed using the Mann-Whitney test for variables with nonnormal distribution and the *t* test for those with normal distribution. Mediation analyses were performed by the imputation-based approach using an R package medflex.^13^ The exposure-mediator interactions were evaluated in the natural effect models.^13^ We used R version 3.4.4 (R Project for Statistical Computing) for the statistical analyses. Statistical significance was determined using 2-sided *P* < .10^14^ for interaction and *P* < .05 for other analyses.

## Results

### Cohort Description

Among 1488 patients, the mean (SD) age was 63.5 (9.9) years and 1161 patients (78.0%) were men. The median (IQR) pre-PCI and post-PCI FFR were 0.71 (0.62-0.76) and 0.88 (0.83-0.92), respectively (Table 1). Gray-zone FFR (0.75 to 0.80) and low FFR (<0.75) applied to 495 vessels (33.3%) and 993 vessels (66.7%), respectively. Compared with the gray-zone FFR group, the low FFR group had higher median (IQR) SYNTAX score (10 [7-16] vs 12 [8-18]; *P* < .001) and lower median (IQR) post-PCI FFR (0.89 [0.85-0.93] vs 0.87 [0.82-0.82]; *P* < .001). The comparison of patients with pre-PCI FFR of less than 0.67 and those with pre-PCI FFR of 0.67 or higher is summarized in eTable 1 in the Supplement. eFigure 1 in the Supplement illustrates the distributions of pre-PCI and post-PCI FFR in the total population. Pre-PCI FFR was skewed to the left because of the threshold of 0.80 for PCI indication. The number of patients with post-PCI FFR of 0.80 or less was relatively low and likely represented unsuccessful PCI.

**Table 1.** Baseline Characteristics

Characteristic	Patients, No. (%)			*P* value
	Total (N = 1488)	Gray zone FFR, ie, pre-PCI FFR, 0.75-0.80 (n = 495)	Low FFR, ie, pre-PCI FFR, <0.75 (n = 993)	
Age, mean (SD), y	63.5 (9.9)	64.7 (9.6)	62.9 (10.1)	.001
Men	1161 (78.0)	380 (76.8)	781 (78.7)	.45
Hypertension	976 (65.6)	327 (66.1)	649 (65.4)	.83
Diabetes	481 (32.3)	163 (32.9)	318 (32.0)	.77
Dyslipidemia	724 (48.7)	241 (48.7)	483 (48.6)	>.99
CKD	46 (3.1)	14 (2.8)	32 (3.2)	.80
Smoking	427 (28.7)	132 (26.7)	295 (29.7)	.25
Previous MI	194 (13.0)	73 (14.7)	121 (12.2)	.19
Vessel				
LAD	1122 (75.4)	362 (73.1)	760 (76.5)	.36
LCx	143 (9.6)	52 (10.5)	91 (9.2)	
RCA	223 (15.0)	81 (16.4)	142 (14.3)	
SYNTAX score, median (IQR)	11 (7-17)	10 (7-16)	12 (8-18)	<.001
Pre-PCI FFR, median (IQR)	0.71 (0.62-0.76)	0.77 (0.76-0.79)	0.66 (0.57-0.71)	<.001
Post-PCI FFR, median (IQR)	0.88 (0.83-0.92)	0.89 (0.85-0.93)	0.87 (0.82-0.92)	<.001

Abbreviations: CKD, chronic kidney disease; FFR, fractional flow reserve; IQR, interquartile range; LAD, left anterior descending coronary artery; LCx, left circumflex coronary artery; MI, myocardial infarction; PCI, percutaneous coronary intervention; RCA, right coronary artery.

During a 2-year follow-up, a total of 82 TVFs were observed; in detail, 12 (14.6%) deaths from cardiovascular causes, 5 (6.1%) nonfatal target vessel–related MIs, and 65 (79.3%) clinically driven target vessel revascularizations were confirmed. eFigure 2 in the Supplement shows a scatterplot of pre-PCI FFR vs post-PCI FFR among patients who did and did not experience 2-year TVF. The incidence of TVF was 51 of 637 patients (8.0%) with pre-PCI FFR of less than 0.75 and post-PCI FFR of less than 0.90, 14 of 356 patients (3.9%) with pre-PCI FFR of less than 0.75 and post-PCI FFR of 0.90 or greater, 9 of 275 patients (3.3%) with pre-PCI FFR of 0.75 or greater and post-PCI FFR of less than 0.90, and 8 of 220 patients (3.7%) with pre-PCI FFR of 0.75 or greater and post-PCI FFR of 0.90 or greater.

### Associations Between Exposure, Mediator, and Outcome

eTable 2 in the Supplement shows the associations of pre-PCI FFR, post-PCI FFR, and TVF. In adjusted models, the associations of pre-PCI FFR of less than 0.75 with post-PCI FFR of less than 0.90 and of pre-PCI FFR of less than 0.75 and TVF were statistically significant (odds ratio [OR], 1.30; 95% CI, 1.02-1.67; *P* = .04 and OR, 1.86; 95% CI, 1.06-3.28; *P* = .03, respectively). Post-PCI FFR of less than 0.90 was significantly associated with TVF after adjustment of confounders and pre-PCI FFR (OR, 2.09; 95% CI, 1.18-3.70; *P* = .01). Similar associations were observed in the combination of pre-PCI FFR of less than 0.67, post-PCI FFR of 0.80 or less, and TVF.

### Mediation Analysis

Mediation analyses documented the directions of associations consistent with those found in the logistic regression analyses. When pre-PCI FFR of less than 0.75 and post-PCI FFR of less than 0.90 were set as the exposure and mediator, respectively (Table 2), there was evidence of a significant NDE (OR, 1.81; 95% CI, 1.03-3.17; *P* = .04) and TE (OR, 1.87; 95% CI, 1.06-3.27; *P* = .03) of pre-PCI FFR on TVF. In this model, post-PCI FFR of less than 0.90 explained only 4.7% of the association between pre-PCI FFR and TVF, and the NIE was neither significant nor strong (OR, 1.03; 95% CI, 0.98-1.09; *P* = .24).

**Table 2.** Mediation Analysis

Association	Odds ratio (95% CI)^a^	*P* value
**Exposure, pre-PCI FFR <0.75; mediator, post-PCI FFR <0.90**		
Natural direct effect	1.81 (1.03-3.17)	.04
Natural indirect effect	1.03 (0.98-1.09)	.24
Total effect	1.87 (1.06-3.27)	.03
**Exposure, pre-PCI FFR <0.67; mediator, post-PCI FFR ≤0.80**		
Natural direct effect	1.48 (0.91-2.43)	.12
Natural indirect effect	1.15 (1.03-1.29)	.01
Total effect	1.71 (1.06-2.75)	.03

Abbreviations: PCI, percutaneous coronary intervention; FFR, fractional flow reserve.

^a^ Odds ratios represent the association of low pre-PCI FFR on 2-year target vessel failure.

When pre-PCI FFR of less than 0.67 and post-PCI FFR of 0.80 or less were used as the exposure and mediator, respectively, no significant NDE was observed between pre-PCI FFR and TVF (OR, 1.48; 95% CI, 0.91-2.43; *P* = .12). The NIE was statistically significant but not strong (OR, 1.15; 95% CI, 1.03-1.29; *P* = .01). The mediator, post-PCI FFR, explained 26.1% of the association of pre-PCI FFR with TVF.

The findings of natural effect models with exposure-mediator interaction are shown in eTable 3 in the Supplement. The exposure-mediator interaction was significant in the model with pre-PCI FFR of less than 0.67 as the exposure and post-PCI FFR of 0.80 or less as the mediator (*P *for interaction = .06). In this model, the OR of the pure direct effect (ie, the NDE without the exposure-mediator interaction) was larger than the pure indirect effect, but only the latter was significant (pure direct effect: OR, 1.51; 95% CI, 0.94-2.42; *P* = .09; pure indirect effect: OR, 1.28; 95% CI, 1.06-1.54; *P* = .009). Total indirect effect considering the exposure-mediator interaction was not significant (OR, 1.04; 95% CI, 0.93-1.17; *P* = .48).

### Sensitivity Analysis

**Figure 2.**
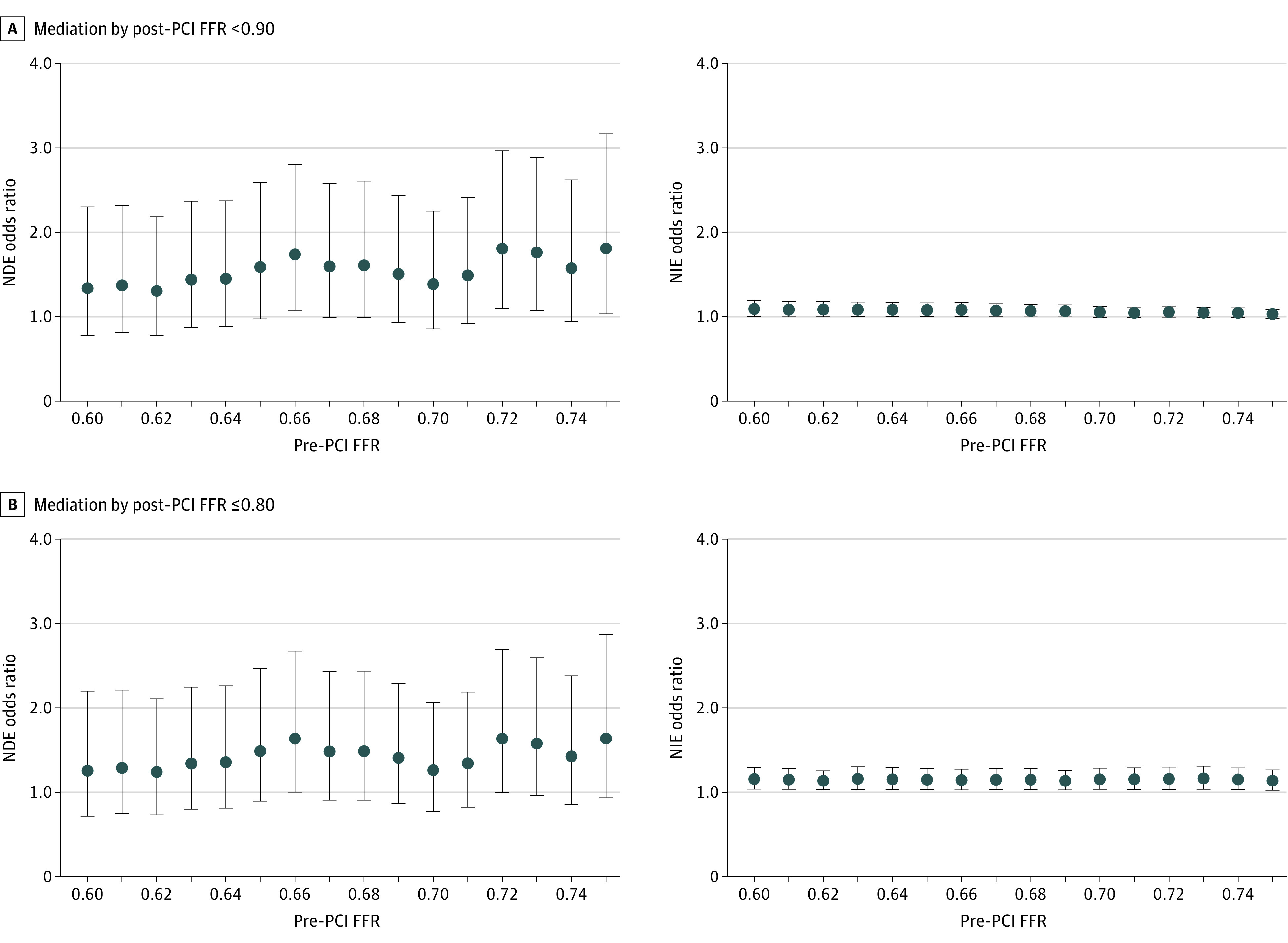
Mediatory Associations of Post–percutaneous Coronary Intervention (PCI) Fractional Flow Reserve (FFR) With Target Vessel Failure at Several Cutoffs of Pre-PCI FFR Dots and bars correspond to the point estimates and 95% CIs.

Mediation by post-PCI FFR on TVF were assessed in several cutoffs of pre-PCI FFR for defining the exposure. When either post-PCI FFR of less than 0.90 (Figure 2A) or of 0.80 or less (Figure 2B) was used as the mediator, the NDE was stronger and had higher variance compared with NIE. The NIE was slightly larger when post-PCI FFR of 0.80 or less was used as the mediator compared with post-PCI FFR of less than 0.90.

When continuous post-PCI FFR was used as the mediator, the results of mediation analyses were not greatly changed; for the exposure of pre-PCI FFR of less than 0.75 (eTable 4 in the Supplement), the NIE was significant in each model (pre-PCI FFR <0.75: OR, 1.15; 95% CI, 1.04-1.13; *P* = .008; pre-PCI FFR <0.67: OR, 1.22; 95% CI, 1.06-1.40; *P* = .004). The magnitude of the NDE was stronger but not statistically significant.

## Discussion

This study evaluated the extent to which post-PCI FFR mediated the association between pre-PCI FFR and TVF and found that the prognostic value of pre-PCI FFR was not appreciably mediated by post-PCI FFR. Our findings highlight that poor prognosis due to progressed atherosclerosis, represented as low FFR, may not be reversed by successful PCI that increases FFR. Therefore, the prognostic information of pre-PCI FFR may mainly reflect the global atherosclerotic burden of the artery, not the extent of the modifiable epicardial stenosis.

**Figure 3.**
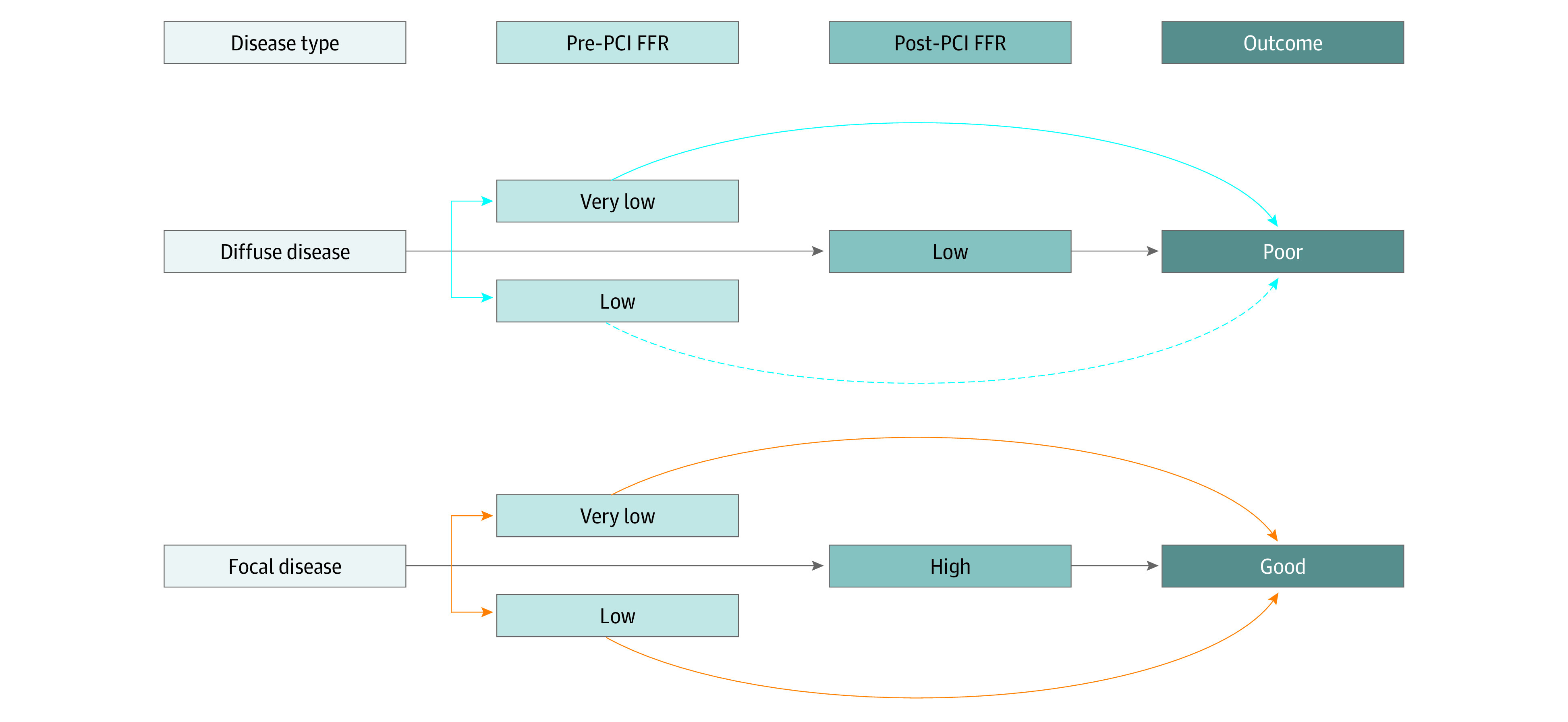
Potential Interpretation of This Study In diffuse disease, percutaneous coronary intervention (PCI) treatment generally results in low post-PCI fractional flow reserve (FFR) and is associated with increased risk of events because of the high residual atherosclerotic burden (gray arrows). In this disease type, pre-PCI FFR may represent the overall atherosclerotic burden; hence, very low pre-PCI FFR was associated with higher incident vessel failure (a blue solid arrow) compared with moderately low pre-PCI FFR (a blue dashed arrow indicating the weaker association). Pre-PCI FFR may have thus the direct effect on TVF, but the post-PCI FFR would be lower regardless of pre-PCI FFR level. In contrast, focal disease generally results in high post-PCI FFR, and future target vessel failure is rare given the limited plaque burden (gray arrows). Therefore, the tightness of the focal stenosis, directly represented by the pre-PCI FFR level, may not be associated with the post-PCI FFR or outcome (orange solid arrows representing similar associations), indicative of little mediation. FFR indicates fractional flow reserve; PCI, percutaneous coronary intervention.

Our observation suggests the clinical implication of the heterogeneity of low FFR as visualized in Figure 3. In diffuse disease, PCI treatment generally results in low post-PCI FFR and is associated with increased risk of events because of the high residual atherosclerotic burden. In this disease type, pre-PCI FFR may well represent the overall atherosclerotic burden; hence, very low pre-PCI FFR was associated with higher incident vessel failure compared with moderately low pre-PCI FFR. Because the post-PCI FFR would be lower regardless of the pre-PCI FFR level, this highlights the minimum mediatory role. In contrast, focal disease generally results in high post-PCI FFR, and future TVF is rare given the limited plaque burden. Therefore, how tight the focal stenosis is, directly represented by the pre-PCI FFR level, may not be greatly associated with the post-PCI FFR or outcome, indicative of little mediation. Therefore, in total, there would be some direct association of pre-PCI FFR with TVF in diffuse disease and little mediation by post-PCI FFR. The direct association of low pre-PCI FFR on future events may be related to integrated information of the target vessel in terms of both epicardial and microvascular condition^15^ and global atherosclerotic burden or ischemia, as is supported by a study showing the link between FFR and global coronary flow reserve.^16^ Thus, additional considerations of coronary flow or microvascular dysfunction with FFR should provide more detailed characterization of the coronary stenosis and accurate prognostic information; for example, coronary flow reserve and coronary flow capacity might provide such information complementary with FFR.^15,17^

The interpretation of this study does not undermine the prognostic value of post-PCI FFR. In reality, low post-PCI FFR was significantly associated with incidence of TVF after adjustment for confounders and pre-PCI FFR (Table 2). This observation implies that post-PCI FFR might hold prognostic information independent of pre-PCI FFR. Slightly stronger mediation by post-PCI FFR of 0.80 or less compared with post-PCI FFR of less than 0.90 also support the significant prognostic value of post-PCI FFR, possibly highlighting unsuccessful PCI and the repeated treatment in lesions with post-PCI of 0.80 or less. In addition, theoretically (as in Figure 3), lower post-PCI FFR could be associated with worse prognosis because it might reflect the nature of underlying coronary stenosis, ie, diffuse or focal disease (represented by the gray straight lines). Accordingly, our observation supports the use of post-PCI FFR as a prognostic surrogate. The prognostic implications of low post-PCI FFR may also represent residual plaque burden masked by a focal tight stenosis or reduced vasomotor function combined with high microvascular resistance, not highlighting the consequences of low pre-PCI FFR.^18^

### Limitations

Our study has several limitations. First, the outcome of TVF could have been subjective because it includes ischemia-driven revascularization and was not adjudicated by a clinical event committee across the 4 registries. Although the study population was relatively large, extensive subgroup analyses were not performed because of the limited incidence of the events. However, this end point is clinically relevant and has been used in previous studies,^10,19^ and our investigators strictly followed the current guidelines, which minimize the subjective nature of the outcome. Second, the study population mainly consisted of Asian individuals, and as such, the generalizability of the study findings might be limited. Third, the exclusion of tight stenosis or severe tortuosity limiting FFR measurement might have led to an underestimation of the extent to which post-PCI FFR might mediate the association between pre-PCI FFR and TVF. Fourth, we cannot rule out the existence of unmeasured confounders, such as small-vessel disease, which a recent paper suggested.^20^ Fifth, our interpretation of the present findings was hypothetical. The availability of data regarding diffuse or focal disease would have further strengthened the interpretation. Furthermore, the fourth assumption of mediation analysis detailed in the Methods section could not be fully satisfied. Certain PCI procedures could be affected by pre-PCI FFR and might confound the association between post-PCI FFR and TVF. Nevertheless, the present study applied a counterfactual framework for the first time we are aware of to assess how much post-PCI FFR might mediate the association between pre-PCI FFR and TVF, and the clinical implication of the analysis is clear.

## Conclusions

In this cohort study, the association of pre-PCI with TVF was not significantly mediated by post-PCI FFR. High risk of vessel failure, represented as low pre-PCI FFR, could not be appreciably reversed by successful PCI that increases the FFR value. Therefore, pre-PCI FFR, as a prognostic marker, may mainly reflect the global atherosclerotic burden, not the extent of the modifiable epicardial stenosis. Although post-PCI FFR is associated with future clinical events, pre-PCI FFR might provide additional prognostic information in terms of overall atherosclerotic burden.

## Notes

Supplement. **eFigure 1. **Distribution of FFR **eFigure 2. **Incidence of TVF in the Study Population, Based on Pre-PCI and Post-PCI FFR **eTable 1. **Baseline Characteristics in Patients Divided according to Pre-PCI FFR of Less Than 0.67 or 0.67 and Greater **eTable 2. **Association of Pre-PCI FFR, Post-PCI FFR, and TVF **eTable 3. **Causal Mediation Analysis Considering Exposure-Mediator Interaction **eTable 4. **Sensitivity Analysis Using Continuous Post-PCI FFR
