# Supplementary material for: Prognostic Value of Prerevascularization Fractional Flow Reserve Mediated by the Postrevascularization Level
Source: JAMA Netw Open. 2020 Sep 30;3(9):e2018162. doi: 10.1001/jamanetworkopen.2020.18162 (PMC7527875; doi:10.1001/jamanetworkopen.2020.18162)
Supplement: Supplement. — eFigure 1. Distribution of FFR eFigure 2. Incidence of TVF in the Study Population, Based on Pre-PCI and Post-PCI FFR eTable 1. Baseline Characteristics in Patients Divided according to Pre-PCI FFR of Less Than 0.67 or 0.67 and Greater eTable 2. Association of Pre-PCI FFR, Post-PCI FFR, and TVF eTable 3. Causal Mediation Analysis Considering Exposure-Mediator Interaction eTable 4. Sensitivity Analysis Using Continuous Post-PCI FFR [file jamanetwopen-e2018162-s001.pdf]

Supplementary Online Content

Hamaya R, Mittleman MA, Hoshino M, et al. Prognostic value of prerevascularization fractional flow reserve mediated by the postrevascularization level. *JAMA Netw Open*. 2020;3(9):e2018162. doi:10.1001/jamanetworkopen.2020.18162

eFigure 1. Distribution of FFR

eFigure 2. Incidence of TVF in the Study Population, Based on Pre-PCI and Post-PCI FFR

eTable 1. Baseline Characteristics in Patients Divided according to Pre-PCI FFR of Less Than 0.67 or 0.67 and Greater

eTable 2. Association of Pre-PCI FFR, Post-PCI FFR, and TVF

eTable 3. Causal Mediation Analysis Considering Exposure-Mediator Interaction

eTable 4. Sensitivity Analysis Using Continuous Post-PCI FFR

This supplementary material has been provided by the authors to give readers additional information about their work.

© 2020 Hamaya R et al. *JAMA Network Open.*

eFigure 1. Distribution of FFR

eFigure 1

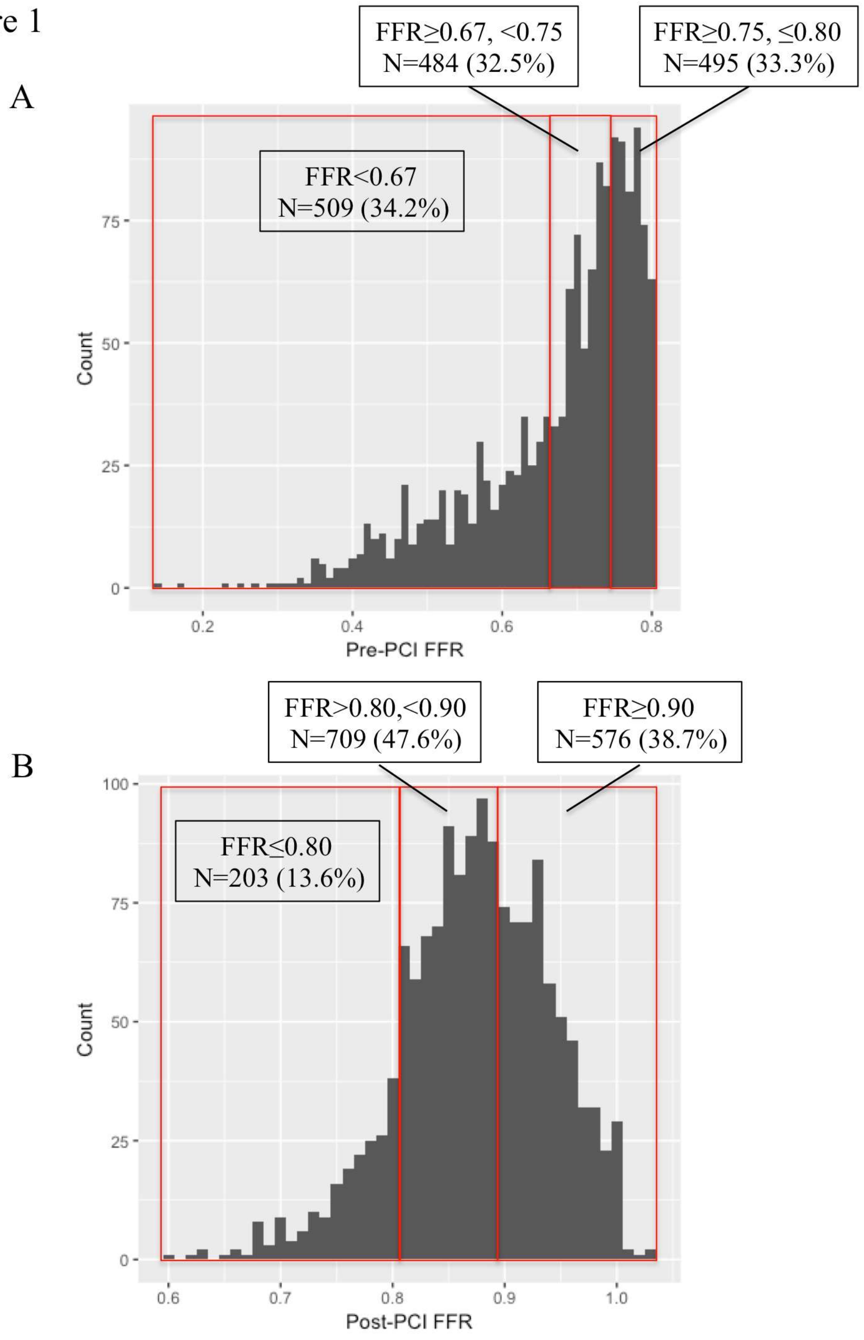

Histograms showing the distribution of pre-PCI FFR (A) and post-PCI FFR (B). Abbreviations: FFR, fractional flow reserve; PCI, percutaneous coronary intervention.

© 2020 Hamaya R et al. *JAMA Network Open.*

eFigure 2. Incidence of TVF in the Study Population, Based on Pre-PCI and Post-PCI FFR

eFigure 2

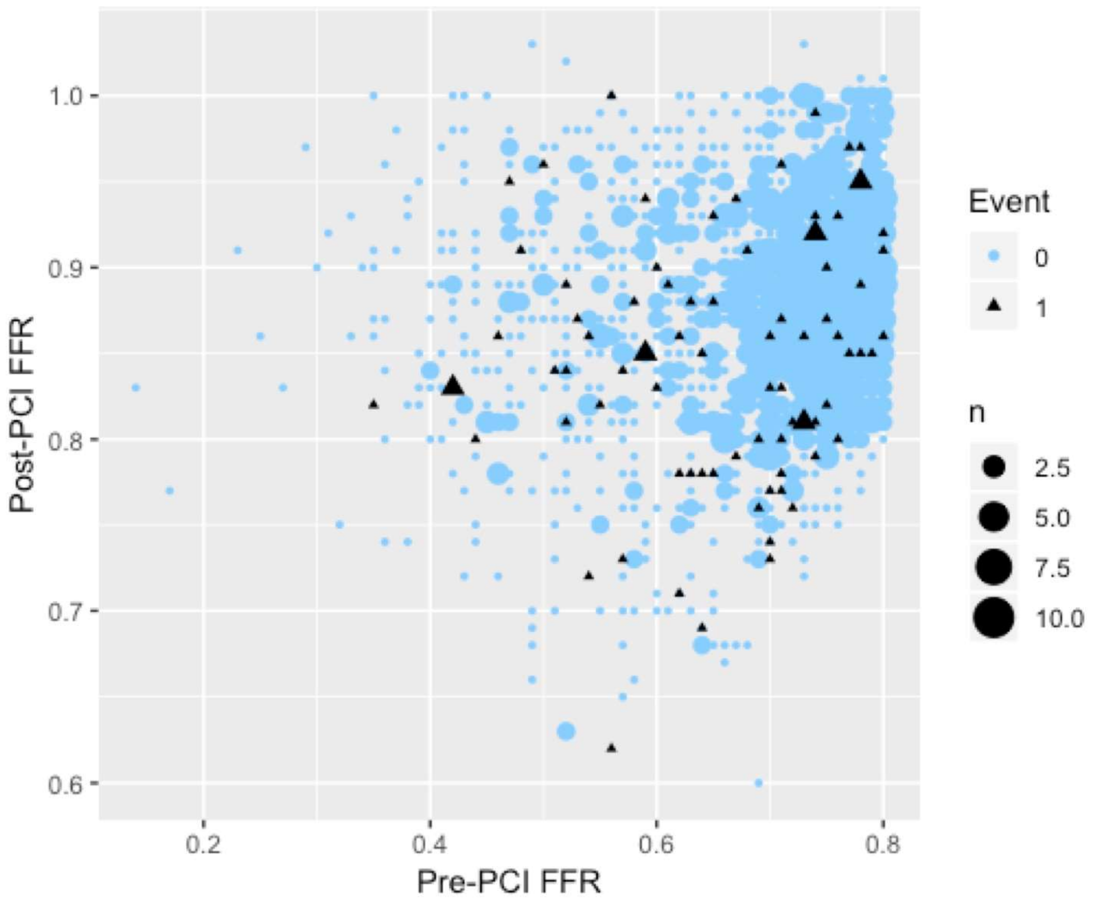

A scatter plot showing the number of participants across the pre- and post-PCI FFR levels. Those who experienced 2-year TVF were indicated as black triangles, and those who did not were represented as blue circles. The size of blue circles represents the number of patients with the exact same pre- and post-PCI FFR. Abbreviations: FFR, fractional flow reserve; PCI, percutaneous coronary intervention; TVF, target vessel failure.

© 2020 Hamaya R et al. *JAMA Network Open.*

eTable 1. Baseline Characteristics in Patients Divided according to Pre-PCI FFR of Less Than 0.67 or 0.67 and Greater

	Pre-PCI FFR: 0.67-0.80	Pre-PCI FFR<0.67	p
n	979	509	
Age	64.4±9.5	61.7±10.5	<0.001
Male	750 (76.6)	411 (80.7)	0.078
Hypertension	653 (66.7)	323 (63.5)	0.23
Diabetes Mellitus	341 (34.8)	140 (27.5)	0.005
Dyslipidemia	499 (51.0)	225 (44.2)	0.015
Chronic kidney disease	32 (3.3)	14 (2.8)	0.70
Smoking	278 (28.4)	149 (29.3)	0.77
Previous MI	132 (13.5)	62 (12.2)	0.53
Vessel (%)			0.97
LAD	738 (75.4)	384 (75.4)	
LCx	93 (9.5)	50 (9.8)	
RCA	148 (15.1)	75 (14.7)	
SYNTAX score	10 [7, 16]	13 [8, 19]	<0.001
Pre-PCI FFR	0.75 [0.71, 0.77]	0.57 [0.49, 0.62]	<0.001
Post-PCI FFR	0.88 [0.84, 0.93]	0.87 [0.81, 0.92]	<0.001

Data are shown as mean±SD, median [IQR], or number (%).

Abbreviations: PCI, percutaneous coronary intervention; FFR, fractional flow reserve; MI, myocardial infarction; LAD, left anterior descending coronary artery; LCx, left circumflex coronary artery; RCA, right coronary artery.

© 2020 Hamaya R et al. *JAMA Network Open.*

eTable 2. Association of Pre-PCI FFR, Post-PCI FFR, and TVF

	Odds Ratio	95% confidence interval	p
Exposure: pre-PCI FFR<0.75, Mediator: post-PCI FFR<0.90			
• *Association of pre-PCI FFR<0.75 and post-PCI FFR<0.90*			
Univariable model	1.43	1.15, 1.78	0.001
Adjusted model*	1.30	1.02, 1.67	0.038
• *Association of pre-PCI FFR<0.75 and TVF*			
Univariable model	1.97	1.14, 3.40	0.015
Adjusted model*	1.86	1.06, 3.28	0.031
• *Association of post-PCI FFR<0.90 and TVF*			
Univariable model	1.77	1.08, 2.92	0.025
Adjusted model1*	2.13	1.21, 3.76	0.009
Adjusted model2‡	2.09	1.18, 3.70	0.012
Exposure: pre-PCI FFR<0.67, Mediator: post-PCI FFR≤0.80			
• *Association of pre-PCI FFR<0.67 and post-PCI FFR≤0.80*			
Univariable model	2.57	1.90, 3.46	<0.001
Adjusted model*	2.58	1.86, 3.58	<0.001
• *Association of pre-PCI FFR<0.67 and TVF*			
Univariable model	1.71	1.10, 2.68	0.018
Adjusted model*	1.71	1.05, 2.77	0.031
• *Association of post-PCI FFR≤0.80 and TVF*			
Univariable model	2.48	1.49, 4.14	0.001
Adjusted model1*	3.16	1.75, 5.72	<0.001
Adjusted model2‡	2.90	1.59, 5.29	0.001

*Models adjusted by confounders: age (years, continuous), sex, hypertension, diabetes mellitus, dyslipidemia, chronic kidney disease, smoking status, prior MI, vessel location, pre-PCI SYNTAX score (quartile), and the original registry (categorical).

‡Models adjusted by confounders and exposures (pre-PCI FFR).

Abbreviations: PCI, percutaneous coronary intervention; FFR, fractional flow reserve; TVF, target-vessel failure.

© 2020 Hamaya R et al. *JAMA Network Open.*

eTable 3. Causal Mediation Analysis Considering Exposure-Mediator Interaction

	Odds ratio	95% confidence interval	p
• *Exposure: pre-PCI FFR<0.75, Mediator: post-PCI FFR<0.90*			
Exposure-mediator interaction			0.14
Pure direct effect	1.59	0.92, 2.74	0.096
Total direct effect	1.72	0.99, 3.00	0.055
Pure indirect effect	1.00	0.92, 1.08	0.99
Total indirect effect	1.09	1.01, 1.17	0.029
Total effect	1.72	1.01, 2.96	0.048
• *Exposure: pre-PCI FFR<0.67, Mediator: post-PCI FFR≤0.80*			
Exposure-mediator interaction			0.059
Pure direct effect	1.51	0.94, 2.42	0.086
Total direct effect	1.23	0.75, 2.01	0.41
Pure indirect effect	1.28	1.06, 1.54	0.009
Total indirect effect	1.04	0.93, 1.17	0.48
Total effect	1.57	0.98, 2.53	0.061

Odds ratios represent the effect of low pre-PCI FFR on 2-year TVF.

Pure direct/indirect effect refers to the natural direct/indirect effect without the exposure-mediator interaction. Total direct/indirect effect means the natural direct/indirect effect considering the exposure-mediator interaction.

Abbreviations: PCI, percutaneous coronary intervention; FFR, fractional flow reserve; TVF, target-vessel failure.

© 2020 Hamaya R et al. *JAMA Network Open.*

eTable 4. Sensitivity Analysis Using Continuous Post-PCI FFR

	Odds ratio	95% confidence interval	p
• *Exposure: pre-PCI FFR<0.75, Mediator: continuous post-PCI FFR*			
Natural direct effect	1.62	0.93–2.83	0.092
Natural indirect effect	1.15	1.04–1.28	0.008
Total effect	1.86	1.06–3.26	0.029
• *Exposure: pre-PCI FFR<0.67, Mediator: continuous post-PCI FFR*			
Natural direct effect	1.40	0.85–2.30	0.19
Natural indirect effect	1.22	1.06–1.40	0.004
Total effect	1.71	1.06–2.76	0.028

Odds ratios represent the effect of low pre-PCI FFR on 2-year TVF.

The natural direct effect represents the average effect of low pre-PCI FFR on TVF, had the effect of pre-PCI FFR on post-PCI FFR been blocked so that post-PCI FFR remains as under non-low pre-PCI FFR status. The natural indirect effect represents the estimated effect of low pre-PCI FFR on TVF when controlling pre-PCI FFR while changing post-PCI FFR status from the level it would have been at low pre-PCI FFR. Total effect indicates the overall effect of the exposure on the outcome.

Abbreviations: PCI, percutaneous coronary intervention; FFR, fractional flow reserve; TVF, target-vessel failure.

© 2020 Hamaya R et al. *JAMA Network Open.*
